# Epicardial delivery of autologous atrial appendage micrografts during coronary artery bypass surgery—safety and feasibility study

**DOI:** 10.1186/s40814-017-0217-9

**Published:** 2017-12-20

**Authors:** Annu Nummi, Tuomo Nieminen, Tommi Pätilä, Milla Lampinen, Miia L. Lehtinen, Sari Kivistö, Miia Holmström, Erika Wilkman, Kari Teittinen, Mika Laine, Juha Sinisalo, Markku Kupari, Esko Kankuri, Tatu Juvonen, Antti Vento, Raili Suojaranta, Ari Harjula, The AADC consortium, Ari Harjula, Antti Vento, Juha Sinisalo, Mika Laine, Markku Kupari, Tatu Juvonen, Kari Teittinen, Annu Nummi, Miia Lehtinen, Tuomo Nieminen, Tommi Pätilä, Eero Mervaala, Esko Kankuri, Milla Lampinen, Sari Kivistö, Miia Holmström, Raili Suojaranta, Erika Wilkman, Jari Laurikka, Shengshou Hu, Zhe Zheng, Xie Yanbo

**Affiliations:** 1 Heart and Lung Center, University of Helsinki and Helsinki University Hospital, Helsinki, Finland; 2 Department of Internal Medicine, South Karelia Central Hospital, Lappeenranta, Finland; 3 Pediatric Cardiac Surgery, Children’s Hospital, University of Helsinki and Helsinki University Hospital, Helsinki, Finland; 4 Department of Pharmacology, Faculty of Medicine, University of Helsinki, Helsinki, Finland; 5 HUS Medical Imaging Center, Radiology, University of Helsinki and Helsinki University Hospital, Helsinki, Finland; 6 Department of Anesthesiology and Intensive Care, University of Helsinki and Helsinki University Hospital, Helsinki, Finland

**Keywords:** Autologous micrografts, Heart failure, Coronary artery bypass surgery, Cell therapy, Atrial appendage, Epicardial cell delivery

## Abstract

**Background:**

The atrial appendages are a tissue reservoir for cardiac stem cells. During on-pump coronary artery bypass graft (CABG) surgery, part of the right atrial appendage can be excised upon insertion of the right atrial cannula of the heart-lung machine. In the operating room, the removed tissue can be easily cut into micrografts for transplantation. This trial aims to assess the safety and feasibility of epicardial transplantation of atrial appendage micrografts in patients undergoing CABG surgery.

**Methods/design:**

Autologous cardiac micrografts are made from leftover right atrial appendage during CABG of 6 patients. Atrial appendage is mechanically processed to micrografts consisting of atrial appendage-derived cells (AADCs) and their extracellular matrix (ECM). The micrografts are epicardially transplanted in a fibrin gel and covered with a tissue-engineered ECM sheet. Parameters including echocardiography—reflecting cardiac insufficiency—are studied pre- and post-operatively as well as at 3 and 6 months of the follow-up. Cardiac functional magnetic resonance imaging is performed preoperatively and at 6-month follow-up. The primary outcome measures are patient safety in terms of hemodynamic and cardiac function over time and feasibility of therapy administration in a clinical setting. Secondary outcome measures are left ventricular wall thickness, change in the amount of myocardial scar tissue, changes in left ventricular ejection fraction, plasma concentrations of N-terminal pro-B-type natriuretic peptide (NT-proBNP) levels, New York Heart Association class, days in hospital, and changes in the quality of life. Twenty patients undergoing routine CAGB surgery will be recruited to serve as a control group.

**Discussion:**

This study aims to address the surgical feasibility and patient safety of epicardially delivered atrial appendage micrografts during CABG surgery. Delivery of autologous micrografts and AADCs has potential applications for cell and cell-based gene therapies.

**Trial registration:**

ClinicalTrials.gov Identifier: NCT02672163. Date of registration: 02.02.2016

## Background

Loss of functional myocardium after, for example, an ischemic insult instigates a need for the remaining tissue to undergo various levels of remodeling to compensate for the declining pumping efficacy. Dead tissue is replaced by non-contractile fibrous scar, while hypertrophy and dilatation may be prominent in other parts of the myocardium struggling to compensate for the lost pumping function. Despite improved drug therapies, many patients develop heart failure over time [1]. The prognosis of heart failure is poor; 4-year survival is less than 50%, and 40% of patients are dead or readmitted to hospital within the first year [2, 3]. Long-term survival has increased due to revascularization techniques such as coronary artery bypass grafting (CABG). Unfortunately, 25–30% of the operated patients do not respond to revascularization surgery by improved perfusion. These patients are thus in much higher risk of heart failure and death [4]. Economically, heart disease exerts a severe burden worldwide that is accentuated with population aging. In 2010, for example, heart disease cost the USA $316 billion [5].

During the last decade, cell-based therapies have entered the clinic with a new hope as therapy for heart failure. Several cell transplantation strategies have been evaluated in experimental as well as in clinical trials [6-8]. Consistent improvement in cardiac function has been reported in studies that have used cells of cardiac origin as the therapeutic cell population [9, 10]. Moreover, when patient’s autologous cells are used, the immune system is not activated to reject the cells, which are thus retained longer after transplantation. It has also been shown that enhancing vascular supply of nutrients together with cellular adherence to extracellular substrata further promotes the survival of transplanted cells [11].

In conjunction with revascularization surgery, the aim for cell therapy is to improve the functional recovery of the myocardial scar by restoring the cellular loss of the infarcted area. The cells for therapy need to be able to give rise to all structures of the myocardium. In principle, this means that the therapeutic cells must be capable of forming cardiac muscle (cardiomyocytes), supporting extracellular matrix (fibroblasts) and vasculature (endothelial and smooth muscle cells).

Cardiac-derived stem cells have higher potential to develop along cardiac lineage [7] and differentiate into cardiac myocytes [12] in vivo than the cells derived from other organs. Cardiac stem cells are self-renewing and multipotent, and they have high capacity to form clones and also enhance the differentiation of other progenitor cells to e.g. endothelial and smooth muscle cells. At least six types of stem cells have been isolated and characterized from the heart: Sca-1 cells [13], c-kit cells [14], islet-1 cells [15], cardiac side population cells [16], cardiosphere-derived cells [17], and ALDH(+) (aldehyde dehydrogenase) cells [18]. Cardiac stem cells can be found in abundance in the right atrial appendage [18, 19, 20] which thus represents an appealing source of tissue for cell therapy use.

The efficacy of cell therapy is decreased by the high rate of cell apoptosis soon after the transplantation. Numerous studies have focused on the different routes of the cell delivery. The most investigated approaches are intracoronary and intramyocardial injections and epicardial delivery. Animal models have proven the epicardial delivery to have advantages in securing adequate cell engraftment when compared to any kind of cell injections [21, 22].

Overall, cell-based therapies (by any delivery route) are regarded as safe to the patients [23]. For example, they have been shown to improve left ventricular function [23–26], quality of life, and physical activity tolerance [23, 25, 27]. However, further evidence on safety and feasibility of the epicardial delivery of cardiac stem cells is required.

The primary objective of this trial is to evaluate the clinical feasibility of intraoperative harvesting, processing, and transplantation of autologous atrial appendage tissue micrografts in terms of success in completing the delivery of the transplant to the myocardium in conjunction with cardiac surgery in the operating room as well as in terms of time restrictions associated with both transplant processing and cardiac surgery. Our primary objectives, in terms of safety, are to evaluate acute and 6-month cardiovascular parameters including rhythm, cardiac function, and need for vasoactive or inotropic medication. Our secondary objective is to obtain an initial insight into the therapeutic effect of atrial appendix micrograft transplantation as measured by baseline and 6-month follow-up cardiac functional MRI.

## Methods/design

### Patient selection

In all, 26 patients from the Helsinki University Hospital, Finland, will be recruited to the study in chronological order by the head researcher. Similar to our previous clinical cell therapy trial [28–31], patients of either gender will be initially evaluated for participation by the cardiologists. Patients with heart failure and scheduled for elective CABG are considered eligible for participation in the trial if they meet the inclusion criteria. The criteria for inclusion and exclusion of the patients are presented in Table 1. The first six patients will be recruited to the atrial appendage-derived cell (AADC) arm. Each patient is given both oral and written information about the trial, and the patient’s written informed consent is required for participation and obtained by the head researcher. After scheduling to surgery, patients will undergo a period of at least 4 to 8 weeks for optimization of drug therapy while waiting for the elective operation.

**Table 1:** Inclusion and exclusion criteria for patients enrolled in the study

Criteria for eligibilty
Inclusion criteria
1 Stable coronary artery disease filling the criteria for bypass surgery 2 Age between 18 and 75 years 3 Informed consent obtained 4 LVEF between ≤ 45 and ≥ 20% 5 NYHA class II–IV heart failure symptoms
Exclusion criteria
1 Heart failure due to LV outflow tract obstruction 2 History of life-threatening ventricular arrhythmias or resuscitation, a condition possibly repeating, or an implantable cardioverter-defibrillator 3 Stroke or other disabling condition within 3 months before screening 4 Severe valve disease or scheduled valve surgery 5 Renal dysfunction (GFR < 84 ml/min/1.73 m) 6 Other disease limiting life expectancy 7 Contraindications for coronary angiogram or MRI 8 Participation in some other clinical trial

*LVEF* left ventricular ejection fraction, *NYHA* New York Heart Association, *LV* left ventriculum, *GFR* glomerular filtration rate

Echocardiography (ECHO), quality of life (QoL) questionnaire (standardized SF-36 health survey questionnaire), New York Heart Association (NYHA) class, basic laboratory tests, and N-terminal pro-B-type natriuretic peptide (NT-proBNP) from blood are all evaluated at baseline as well as at 3 and 6 months of the follow-up. Blood RNA and EDTA plasma samples are taken at the pretrial and at 6 months of the follow-up to search markers for prognostic factors. Cardiac MRI is performed at the pretrial and at 6 months of the follow-up.

To determine the safety of the procedure, the next 20 patients who meet the same inclusion and exclusion criteria and are scheduled for elective CABG operation are recruited to the study as control patients. These patients are treated according to the normal hospital protocol, without the extracellular matrix (ECM) sheet and any additional imaging, examination, or blood tests required for the AADC therapy group. Written consent is obtained also from the control patients.

### Cell isolation

**Fig. 1:**
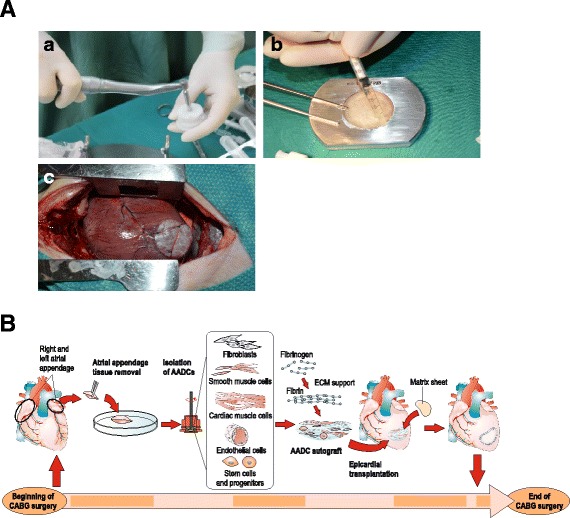
**a** Preparing the AADC-sheet. (A) The atrial appendage tissue is processed with cell therapy tissue homogenizer (Rigenera-system). (B) The micrografts are secured to extracellular matrix sheet (Cormatrix®) by using a fibrin sealant (Tisseel™). (C) The AADC sheet is placed to the myocardium in the location of infarction scar (animal model). **b** Administration of therapy during CABG surgery. Figure reproduced from our article by Lampinen et al. (Current Gene Therapy, 2015)

During CABG surgery, autologous cardiac cells are harvested from a leftover right atrial appendage tissue that is removed for insertion of the heart-and-lung machine. This material serves a safely removable source for cardiac cells. However, quality of the tissue varies greatly between the patients: atrial fibrillation and dilatation as well as diabetes mellitus and obesity may cause accumulation of connective and adipose tissue [32–34], which decrease the quality of the appendage tissue. Also, the size of appendage differs between patients according to age and gender. To standardize the amount of tissue removed for harvesting, the size of removable appendage is 5 × 10 mm and the weight from 600 to 800 mg. The harvested tissue is processed on site in the operating room using a cell therapy tissue homogenizer (Rigenera System, HBW s.r.l., Turin, Italy) (Fig. 1a (A)) [35]. The device is CE-marked and in clinical use for isolation of therapeutic cells from a skin biopsy to treat hard-to-heal or chronic wounds. This system enables extraction of 5–10 millions of single cells per gram of atrial appendage tissue. In addition to these single cells that can be counted, the mixture contains a vast number of cell clusters, i.e., micrografts containing many interconnected cells attached to their extracellular matrix, raising the estimated cell number to reach at least 60 million viable cells per gram of tissue. The isolation of the cells can be performed with ease and strict adherence to sterility in the operating room by a nurse who has received basic training in using the instruments. Initial training was carried out in a cell culture laboratory, and the protocol to accompany CABG surgery was further trained in a large animal model. This cell isolation technology is a straightforward approach for administering autologous cell therapy in a routine and safe manner during surgical procedures. The system utilizes a sterile, single-use tissue homogenizer surface to generate the micrografts.

The isolated AADC micrografts are applied in within cardioplegia suspension as a composite graft on the damaged myocardium using the epicardial transplantation technique. Firstly, the suspension of autologous, freshly isolated AADCs are applied onto the extracellular matrix sheet (Cormatrix® ECM™ Technology, Cormatrix Cardiovascular Inc., Atlanta, GA, USA). Fibrin sealant (Tisseel™, Baxter Healthcare Corp. Westlake Village, CA, USA), routinely used in surgery for hemostasis and tissue sealing, will be added into the cell suspension in order to secure the adherence of the suspension to the matrix (Fig. 1a (B)). Then, the matrix sheet is placed on top of the damaged myocardium. Administration of the transplant is presented in Fig. 1b. The Cormatrix® readily attaches to the tissue but will be further secured to the myocardial surface by suturing (Fig. 1a (C)).

### Therapy administration

A standard CABG operation is performed under cardiopulmonary bypass and mild hypothermia. The operations take place under cardiac arrest and cardioplegia protection. After completion of the bypass anastomoses, each patient receives, under cardiac arrest, a Cormatrix® patch containing autologous cells in physiological saline and fibrin sealant on the infarction area. The area of application is selected before surgery by using the pre-CABG MRI imaging data. The therapy application procedure is carefully photographed during each surgery, and the treatment administration site is specified in patient documents for analysis.

### Clinical cardiac MRI

Cardiac MRI is performed with a 1.5T Avanto fit scanner and phase array cardiac coil (Siemens, Erlangen, Germany). Images will be obtained with electrocardiography (ECG) gating and during breath-holding. LV structure and function are imaged by a standardized MRI protocol [36]. TrueFISP cine series is obtained at the vertical and horizontal long axes for scout to line up short-axis images. The stack of short-axis images is obtained from the mitral valve plane through the apex. To detect the myocardial scar, late gadolinium enhancement (LGE) is imaged with a 2D-segmented inversion recovery gradient ECHO sequence 12 to 20 min after Dotarem® (279.3 mg/ml; dose 0.2 mmol/kg) injection. LGE images are obtained at the same views and slice/gap thickness as cine imaging.

### End-point measures

Table 2 lists the outcome measures of the trial. Specifically, the primary outcome measures are patient safety in terms of hemodynamic and cardiac function and feasibility of the therapy administration in a clinical setting. Hemodynamics is evaluated during the operation and stay at the intensive care unit (ICU) by the need for vasoactive medication and success of weaning from cardiopulmonary bypass and respirator. Postoperative hemodynamic criteria for assessing safety are cardiac index, hemoglobin, SvO_2_, serum potassium level, and blood glucose level. Cardiac function is evaluated also during the operation by transesophageal ECHO and during the stay at ICU by transthoracical ECHO as well as telemetric monitoring of rhythm. Feasibility is evaluated by the success in completing the delivery of the cell sheet to the myocardium, waiting times in minutes for either the cell sheet or the heart after finishing anastomoses, and the success in closing the right atrial appendage by purse string suture without additional sutures or patching.

**Table 2:** Primary and secondary outcome measures

Primary outcome measures	Secondary outcome measures
Safety	Preliminary efficacy
For assessing hemodynamics during the operation and at the intensive care unit	For assessing cardiac function and remodeling as measured by MRI
1. Need for vasoactive medication	1. Left ventricular wall thickness
2. Cardiac index in l/min/m^2^	2. Change in the amount of myocardial scar tissue
3. Hemoglobin in g/l	3. Change in left ventricular ejection fraction
4. Oxygen saturation in the pulmonary arterial blood (SvO_2_) in %	4. Change in movement and diastolic function of left ventricular wall
5. Serum potassium level in mmol/l	Others
6. Blood glucose level in mmol/l	1. Plasma concentrations of N-terminal pro-B-type natriuretic peptide (NT-proBNP) levels
For assessing cardiac function during and after the operation by echocardiogram	2. New York Heart Association class
7. Left ventricular ejection fraction (EF) in %	3. Days in hospital
8. Pericardial effusion in mm	4. Changes in the quality of life measured by questionnaire
For assessing cardiac function after the operation	
9. Telemetric monitoring of rhythm	
Feasibility	
1. Success in completing the delivery of the transplant to the myocardium	
2. Waiting time in minutes for the finished transplant to be placed on the myocardium after doing all the required anastomoses	
3. Waiting time in minutes for the heart after doing all the anastomoses and before the transplant is finished	
4. Closing the right atrial appendage after removing the standardized tissue piece for preparing the transplant	

The secondary outcome measures are change in LV wall thickness and movement and diastolic function as measured by MRI, change in the amount of myocardial scar tissue as measured by MRI, local changes in systolic and diastolic measures as estimated by ECHO, changes in left ventricular ejection fraction (LVEF), pro-BNP level, NYHA class, hospitalization or the days in hospital, and QoL.

**Fig. 2:**
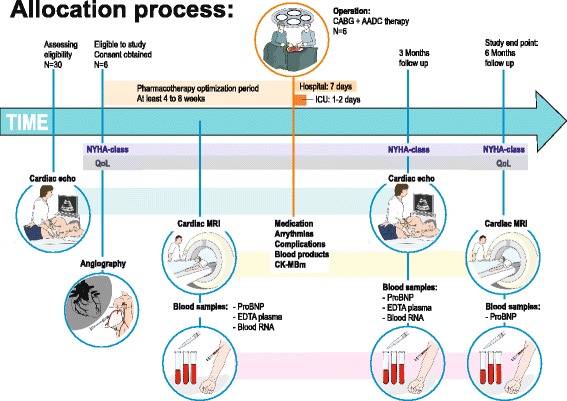
Timeline of the study indicating enrollment and follow-up as well as timing of laboratory tests, MRI, and echocardiographic imaging modalities

Figure 2 presents the timeline of the study indicating enrollment and follow-up as well as timing of laboratory tests, MRI, and echocardiographic imaging for each patient.

### Statistical analysis

Statistical methodology as published previously by Pätilä et al. [28] will be utilized. Briefly, the Mann-Whitney *U* test will be used for non-parametric continuous variables and results will be reported as median with interquartile range (IQR). Categorical variables will be analyzed using Fisher’s exact test. Intra-observer variability will be assessed using the Bland-Altman method. Testing for *p* values will be carried out two-sided with *p* < 0.05 considered statistically significant. Results should be treated as preliminary due to the small sample size and will be presented with 95% confidence intervals. A sample size of 26 has been shown to be sufficient in our previous studies to demonstrate safety. Computation will be achieved with PASW Statistics (IBM Inc., Armonk, NY) or equivalent software.

### Safety considerations

The patients are monitored, and their cardiac function is closely evaluated under the hospitalization period. In case of any abnormalities, the head researcher will be immediately informed and the study will be halted for evaluation of the cause. Continuation of the study will then be further assessed by the head researcher and the clinical panel responsible for the patient care.

## Discussion

The specific aim of the project is to evaluate safety and feasibility of AADC therapy. Patients enrolled to our study have decreased cardiac function and remodeled left ventricle. Therefore, perioperative safety and adverse effect-free long-term survival are the main concerns regarding the planned procedure and follow-up in our study. The study protocol has been designed keeping also patient convenience in mind. Appendage tissue is harvested during cannulation of the right atrium, and therefore, no additional procedure is needed. Isolation of the cells and preparing the matrix for transplantation is done simultaneously with the CABG operation in the operating room, so the perfusion time and the aorta clamp time are not increased. After the bypass anastomoses, the AADC sheet is placed on the myocardium with three to four sutures allowing the myocardium to contract without interference. Immediately after CABG, the heart is most likely to develop postoperative ischemia due to aorta cross-clamping and following reperfusion. This may change the metabolism of the heart and influence ability of tissues to adapt to the AADC sheet placed on the myocardium. Post-operatively, the patients are closely monitored at the intensive care unit and at the ward in case for any arrhythmias, infection-related decrease in cardiac function, or hemodynamic abnormalities. After the hospitalization period, the patients are followed up at 3 and 6 months for any adverse effects or disturbance in the recovery.

Optimal pharmacotherapy for heart failure and revascularization are crucial, but currently, no medical treatment or surgery can repair the irreversibly infarcted and failing myocardium. Autologous cell therapy offers a way to replace dead cardiac tissue with a viable and functional one. Cell therapies have been demonstrated to be generally safe treatments for heart failure in numerous studies [23, 26, 27]. According to meta-analysis of cell therapy trials, therapies are associated with minimal major intervention-related effects and do not increase the incidence of arrhythmias [37]. The review concluded that cell therapy reduced mortality and re-hospitalization caused by heart failure during long-term follow-up and improved global LVEF, NYHA functional class, proBNP levels, and QoL [37]. On the other hand, some trials reporting results from the myoblast intramyocardial injection have demonstrated increased frequency of arrhythmias compared to epicardial delivery [38–41].

In our previous study, we explored the effects of autologous intramyocardial bone marrow mononuclear cell (BMMC) transplantation in ischemic LV dysfunction [32]. The transplantation was done by injecting autologous BMMCs to the myocardial infarction area intra-operatively. We found no harmful effects of the treatment in any clinically relevant parameters (assessing cardiac function, hemodynamic response, and perioperative myocardial damage) in short- or long-term follow-up [28, 31]. The most important finding in that study was that the BMMC therapy showed significant decrease in the size of local scar on MRI [28], which has been proven to be a major prognostic factor after myocardial infarction [42–44]. The biggest challenge with the method of injecting the cells is the low rate of sustained cells in the graft. This is caused by mechanical leakage [45], poor vascularization of the infarcted area, and apoptosis due to loss of cell anchorage (extracellular matrix) [46]. Compared with the injections, a number of studies have demonstrated that epicardial delivery of the therapeutic cells associates with greater number of retained cells [21] and better graft functionality [22]. Therefore, epicardial delivery of cell-seeded micrografts is the primary choice for the delivery route and the approach in our forthcoming study.

The purpose of this study is to address patient safety and clinical feasibility of the therapy. We will determine the success of this trial based on the primary outcome measures. If the therapy is proved to be safe without any disturbances in patients’ cardiac rhythm, hemodynamics, and cardiac function and the method can be successfully performed simultaneously with bypass surgery, the results of this trial are then further evaluated in succeeding trials for transplant’s therapeutic efficacy. Double-blind randomization and placebo control using the matrix sheet with and without the micrografts will be considered for our future studies.

Our interests has also been combining stem cell therapy with gene therapy and using autologous minimally manipulated micrografts in conjunction with CABG surgery [47–49]. By administrating genetically modified transplanted cells, we are able to repair also faulty genetic tissue of the heart [47]. Even though cell therapy has promising results, studies regarding safety and clinical efficacy require further investigation.

This study on feasibility and safety of autologous atrial micrograft epicardial transplantation has some limitations. The allocation is not randomized, and the patients are recruited to the groups in chronological order. Also, this trial is carried out as a one-center trial, and therefore, the number of eligible patients is small. Although randomization does not occur, we have considered that no selection bias will take place due to low number of patients and because all patients who are eligible and give their consent are included in the study.

In conclusion, given the severe problems associated with heart failure and its increasing high prevalence [50] in the elderly population, it is of utmost importance to devise effective novel evidence-based cell therapies. Epicardial delivery of AADCs with ECM cells represents a novel approach to restore the cellular loss of infarcted myocardium. We believe that AADC therapy administered during CABG surgery will have impact on patient treatment in the future.

### Trial status

Recruitment of the patients started in February 2016.

## Abbreviations

AADC: Atrial appendage-derived cell
ALDH(+): Aldehyde dehydrogenase
BMMC: Bone marrow mononuclear cell
CABG: Coronary artery bypass grafting
Cf-MRI: Cardiac functional magnetic resonance imaging
ECG: Electrocardiography
ECHO: Echocardiography
ECM: Extracellular matrix
EDTA: Ethylenediaminetetraacetic acid
GFR: Glomerular filtration rate
ICD: Implantable cardioverter-defibrillator
LGE: Late gadolinium enhancement
LVEF: Left ventricular ejection fraction
NT-proBNP: N-terminal pro-B-type natriuretic peptide
NYHA: New York Heart Association
QoL: Quality of life
RNA: Ribonucleic acid
